# Disturbances in Hypothalamic-Pituitary-Adrenal Axis and Immunological Activity Differentiating between Unipolar and Bipolar Depressive Episodes

**DOI:** 10.1371/journal.pone.0133898

**Published:** 2015-07-21

**Authors:** Karlijn Becking, Annet T. Spijker, Erik Hoencamp, Brenda W. J. H. Penninx, Robert A. Schoevers, Lynn Boschloo

**Affiliations:** 1 University of Groningen, University Medical Center Groningen, Interdisciplinary Center Psychopathology and Emotion regulation (ICPE) and University Center Psychiatry (UCP), Groningen, The Netherlands; 2 PsyQ Rijnmond, Department of Mood Disorders, Rotterdam, The Netherlands; 3 Leiden University, Institute of Psychology, Leiden, The Netherlands; 4 VU University Medical Center, Department of Psychiatry and EMGO+ Institute for Health and Care Research, Amsterdam, The Netherlands; 5 Leiden University Medical Center, Department of Psychiatry, Leiden, The Netherlands; UTHSCSH, UNITED STATES

## Abstract

**Introduction:**

Differentiating bipolar depression (BD) from unipolar depression (UD) is difficult in clinical practice and, consequently, accurate recognition of BD can take as long as nine years. Research has therefore focused on the discriminatory capacities of biomarkers, such as markers of the hypothalamic-pituitary-adrenal (HPA) axis or immunological activity. However, no previous study included assessments of both systems, which is problematic as they may influence each other. Therefore, this study aimed to explore whether cortisol indicators and inflammatory markers were a) independently associated with and/or b) showed effect modification in relation to a lifetime (hypo)manic episode in a large sample of depressed patients.

**Methods:**

Data were derived from the Netherlands Study of Depression and Anxiety and comprised 764 patients with a DSM-IV depressive disorder at baseline, of which 124 (16.2%) had a lifetime (hypo)manic episode at the 2-year assessment, or a more recent episode at the 4-year or 6-year assessment. Baseline cortisol awakening response, evening cortisol and diurnal cortisol slope were considered as cortisol indicators, while baseline C-reactive Protein (CRP), Interleukin-6 (IL-6), and Tumor Necrosis Factor Alpha (TNF-α) were included as inflammatory markers.

**Results:**

In depressed men and women, none of the cortisol indicators and inflammatory markers were (independently) associated with a (hypo)manic episode. However, effect modification was found of diurnal cortisol slope and CRP in relation to a (hypo)manic episode. Further analyses showed that depressed men with high levels of diurnal cortisol slope and CRP had an increased odds (OR=10.99, p=.001) of having a (hypo)manic episode. No significant differences were found in women.

**Conclusion:**

Our findings suggest that the combination of high diurnal cortisol slope and high CRP may differentiate between UD and BD. This stresses the importance of considering HPA-axis and immunological activity simultaneously, but more research is needed to unravel their interrelatedness.

## Introduction

Accurate and timely recognition of bipolar disorder (BD) is very low, and receiving the correct diagnosis can take as long as 9 years [1]. Delayed recognition is associated with a more severe clinical course and substantial economic costs [2]. BD is often misdiagnosed as unipolar depression (UD) at first [3]; around 40% of BD patients were initially diagnosed with a unipolar major depressive episode [4]. This is because approximately half of BD patients present with a major depressive episode as their first mood episode [5], and patients often do not report their manic symptoms to their physicians [6]. Previous studies have identified some predictors of conversion from UD to BD, such as male gender, childhood trauma, severity of depressive symptoms and co-occurring manic symptoms during depression [7,8]. However, in clinical practice depressive episodes in the course of a bipolar or unipolar disorder are almost identical and difficult to differentiate [9].

To elucidate underlying mechanisms involved in the differentiation of the mood episode in a unipolar or bipolar mood disorder, it may be of interest to examine whether biological markers found to be related to mood disorders can discriminate between UD and BD. Up to now, biological disturbances both in the regulation of the hypothalamic-pituitary-adrenal axis (HPA-axis) as well as immunological activity are believed to play a key role in UD [10,11] and BD [12,13]. Previous studies have, for example, found higher cortisol levels and higher levels of inflammatory markers in BD patients [14–18], as well as UD patients [19–23] compared to healthy controls. Studies directly comparing unipolar and bipolar depression are scarce, but the few available studies suggest that HPA-axis disturbances [24] and immune dysregulation [25] are more pronounced in bipolar depression. Furthermore, in the Netherlands Study of Depression and Anxiety (NESDA) various subtypes of unipolar and bipolar depression have been examined. For example, Jabben et al. showed that bipolar spectrum patients had a higher diurnal cortisol slope compared to patients with UD [26]. In a separate paper, Becking et al. recently showed that depressed patients with co-occurring manic symptoms did not significantly differ from depressed patients without manic symptoms in any of the inflammatory markers, but associations were found with the onset of manic symptoms after 2 years follow-up [27].

Most studies that focussed on the associations of these systems with mood disorders included only assessments of either HPA-axis or immunological activity [10,13,16,20]. Consequently, it was not possible to examine the interrelatedness of these systems. This might be problematic, as the systems influence each other. For example, hyperactivity of the HPA-axis, with an underlying glucocorticoid resistance, leads to an increased inflammatory response on a cellular level [28,29]. Similarly, increased pro-inflammatory cytokine levels can lead to inhibition of the glucocorticoid receptor function and directly activate the HPA-axis in the brain [30]. Although convincing evidence has demonstrated the interrelatedness of the systems, it is unclear as to how they influence each other in their relations with mood disorders [30,31].

Another complicating factor in identifying factors that could differentiate between unipolar and bipolar depression, is the influence of gender differences on phenomenology and biology of both mood disorders. Gender differences exist in prevalence [32], symptoms and treatment of mood disorders [33]. For example, there is an increased prevalence of unipolar depression in women, which is associated with fluctuations in gonadal hormones [34]. This may point to differences in biological pathophysiological processes. Therefore, it has been advocated that gender should not merely be analyzed as a covariate, but men and women should be analyzed separately to really understand gender differences in the relation of the HPA-axis [35] as well as the immune system [10] with mood disorders.

To our knowledge, NESDA is the first study that assessed both cortisol indicators and inflammatory markers in a large sample of depressed men and women and would, thus, be suitable for examining the interrelatedness of the systems in relation to the presence of a lifetime (hypo)manic episode. Data were, therefore, derived from this study and we aimed to explore:

1. Whether cortisol indicators and inflammatory markers were independently associated with a lifetime (hypo)manic episode
2. Whether cortisol indicators and inflammatory markers showed effect modification in relation to a lifetime (hypo)manic episode

Because of reasons mentioned earlier, the results are shown for men and women separately. In addition, we were able to explore the role of potential confounders, such as sociodemographics, sampling and health factors.

## Materials and Methods

### Study sample

Data were derived from the Netherlands Study of Depression and Anxiety (NESDA), an ongoing cohort study (N = 2981, age 18–65 years) including 2329 patients with a lifetime depressive and/or anxiety disorder as well as 652 healthy controls. Participants were recruited from the community (19%), general practice (54%), and specialized mental health care (27%). Participants with insufficient command of the Dutch language, or patients with a primary clinical diagnosis of bipolar disorder, obsessive-compulsive disorder, severe substance use disorder, or psychotic disorder were excluded from the study. The study protocol was approved centrally by the Ethical Review Board of the VU University Medical Centre and subsequently by the Ethical Review Boards of the other participating centers (Leiden University Medical Center and University Medical Center Groningen). Written informed consent was obtained from all participants (see [36] for a detailed description of NESDA). So far, NESDA included face-to-face assessments at baseline and 2-year (N = 2596, 87.1%), 4-year (N = 2402, 80.6%), and 6-year (N = 2256, 75.7%) follow-up.

### Unipolar versus bipolar depressive episodes

For the present study, we selected patients with a current DSM-IV depressive episode (N = 1158; <6 months MDD or dysthymia) at baseline, as assessed with the Composite International Diagnostic Interview (CIDI, version 2.1) [37]. Unfortunately, the CIDI section on (hypo)manic episodes was not assessed at baseline, and consequently it was only possible to determine which patients met criteria for a DSM-IV (hypo)manic episode at follow-up assessments after 2 years (lifetime diagnosis), 4 years (diagnosis since the last interview) or 6 years (diagnosis since the last interview). Of our sample of 1158 depressed patients at baseline, 6 (0.5%) patients had missing information on cortisol indicators and inflammatory markers. Of these 1152 depressed patients, 388 (33.7%) did not have valid data at the 6-year follow-up assessment of a (hypo)manic episode and were therefore excluded for the current analyses. This resulted in a total sample of 764 depressed patients, of which 124 (16.2%) had, and 640 (83.8%) did not have a lifetime (hypo)manic episode at one of the follow-up assessments. The CIDI has proven to be highly reliable and valid for diagnosing unipolar depressive episodes as well as manic episodes [38,39], and in a lesser extent for diagnosing hypomanic episodes [40]. However, Regeer et al. showed that the validity of the CIDI was limited when compared with (hypo)manic episode diagnosis established with the the Structured Clinical Interview for DSM (SCID).

### Cortisol indicators

Baseline cortisol was sampled through saliva using Salivettes (Starstedt AG, Numbrecht, Germany), which is a reliable and minimally intrusive way for assessing the active, unbound form of cortisol. Participants were instructed to collect saliva at seven time points on a regular working day (median time since the assessment of psychopathology and inflammation: 9 days). For the present study, we considered four cortisol indicators using six time points (T1-T6). Based on sampling points at awakening (T1) and 30 (T2), 45 (T3) and 60 (T4) minutes later, two indicators of the Cortisol Awakening Response (CAR) were calculated using a trapezoid formula [41]. The *area under the curve with respect to the ground* (AUCg) estimates the total cortisol exposure during the CAR and provides an indication of overall cortisol level throughout the day. The *area under the curve with respect to the increase* (AUCi) is a measure of the dynamic of the CAR and provides an indication of the sensitivity of the HPA-axis, emphasizing changes in cortisol exposure over time [42]. *Evening cortisol* was based on the mean cortisol level at the sampling points of 10 pm (T5) and 11 pm (T6). In addition, the *diurnal cortisol slope* was calculated as a measure for the mean decline per hour ([cortisol T1 minus cortisol T6] / [time T6 minus time T1]). Detailed information on the assessment of the cortisol indicators in NESDA is provided elsewhere [20].

### Inflammatory markers

Baseline inflammatory markers included *C-reactive Protein* (CRP), *Interleukin-6* (IL-6), and *Tumor Necrosis Factor Alpha* (TNF-α). Fasting blood samples of the participants were obtained at approximately 8 a.m. at the day of the psychiatric interview and kept frozen at -80°C. CRP and IL-6 were assayed at the Clinical Chemistry department of the VU University Medical Center (Amsterdam, the Netherlands). High-sensitivity CRP plasma levels were measured in duplicate by an in-house ELISA based on purified protein and polyclonal anti-CRP antibodies (Dako, Glostrup, Denmark), while plasma IL-6 levels were measured in duplicate by a high-sensitivity enzyme-linked immunosorbent assay (PeliKine Compact ELISA, Sanquin, Amsterdam, the Netherlands). Plasma TNF-α levels were assayed in duplicate at Good Biomarkers Science (Leiden, the Netherlands), using a high-sensitivity solid-phase ELISA (Quantikine HS Human TNF-α Immunoassay, R&D Systems, Minneapolis, MN, USA). Detailed information on the assessment of inflammatory markers in NESDA is provided elsewhere [43].

### Potential confounders

Basic covariates (sociodemographics and sampling factors) and health factors were selected as potential confounders, as they have shown to be associated with the HPA-axis [44], the immune system [45], as well as BD [46–48]. *Sociodemographics* included age, education (in years), and North-European ancestry. *Sampling factors* included awakening time, work status, type of day (weekday vs. weekend) and season (dark vs. light months) of the day of sampling. *Health factors* included smoking, alcohol use, physical activity, sleep, body mass index, cardiovascular disease, diabetes, other chronic diseases, statin use and anti-inflammatory medication use. Information on former or current smoking and alcohol use (no [<1 drink per week], moderate [women: 1–14 drinks, men: 1–21 drinks per week] or heavy [women: >14 drinks, men: >21 drinks per week]) was obtained during the interview. Physical activity was measured with the International Physical Activity Questionnaire [49] in MET-minutes (metabolic equivalent; total effort expended in different activities over one week). Sleep duration was assessed with one item of the 5-item Women’s Health Initiative Insomnia Rating Scale (IRS) [50] and dichotomized (sleep <6 hours, yes or no). BMI was calculated as weight (in kg) divided by height squared (in m^2^). The presence of cardiovascular disease was assessed by self-report and appropriate medication use (Anatomical Therapeutic Chemical [ATC] codes: B01, N02BA01, N02BA15, C01DA, C02, C03, and C07-C10, see [51] for detailed description), while diabetes was considered to be present when the fasting plasma glucose level was >7.0 mmol l^-1^ or the patient used antidiabetic medication (ATC code: A10). Furthermore, we assessed self-reported chronic disease for which patients received treatment, including lung-disease, osteoarthritis or rheumatic disease, cancer, ulcer, intestinal problems, liver disease, epilepsy and thyroid gland disease. Medication included use of statins (ATC codes: C10AA and C10B) and anti-inflammatory medication (ATC codes: M01A, M01B, A07EB and A07EC).

### Statistical analyses

Data were analyzed with SPSS version 22.0 (SPSS, Chicago, IL, USA). Evening cortisol, CRP, IL-6 and TNF-α were ln-transformed to normalize distributions and presented back-transformed in tables. First, characteristics (i.e., sociodemographics, sampling factors, and health factors) of depressed patients were compared across those without versus those with a lifetime (hypo)manic episode using T-tests for continuous variables and Pearson’s Chi-square tests for dichotomous and categorical variables. Second, Pearson’s correlations between all cortisol indicators and all inflammatory markers were calculated to explore their interrelatedness.

Then, separate logistic regression analyses were performed to explore whether each of the cortisol indicators and inflammatory markers were associated with a lifetime (hypo)manic episode in our sample of depressed patients. To determine the *independent associations* of cortisol indicators and inflammatory markers with a lifetime (hypo)manic episode, multivariable analyses were conducted including those indicators and markers that had a p <.10 in the previous analyses. To examine *effect modification* between HPA-axis activity and immunological activity in relation to a lifetime (hypo)manic episode, we tested the interaction terms between all cortisol indicators and all inflammatory markers. If significant (p <.05), the nature of the effect modification was further explored by combining information on the level of the cortisol indicator and inflammatory marker (values in the highest tertile were considered high). Consequently, we could compare the presence of a lifetime (hypo)manic episode across depressed patients with a) high cortisol only, b) high inflammation only, and c) both high cortisol and inflammation, to d) depressed patients with low levels of cortisol and inflammation. All previous analyses were adjusted for basic covariates (sociodemographics and sampling factors). To examine whether significant associations could be explained by health factors, analyses were additionally adjusted for these factors.

## Results

In our sample of depressed patients, 124 patients (16.2%) had a lifetime (hypo)manic episode. The mean age of onset of the (hypo)manic episode was 29 years. 7 patients (5.6%) had their last (hypo)manic episode before baseline measurement. Of the remaining patients, 67 (54.0%) had their last (hypo)manic episode 0–2 years after baseline measurement, 25 (20.2%) after 2–4 years and 25 (20.2%) after 4–6 years. Table 1 shows the characteristics of our sample of depressed patients, comparing those with and without a lifetime (hypo)manic episode. In depressed men, a significant difference was found for years of education, but not any other factor. In women, no significant differences were found. Table 2 shows the correlations across all cortisol indicators and inflammatory markers. Correlations between cortisol indicators and inflammatory markers were low in both depressed men (all r≤.17) and depressed women (all r≤.09), likely reflecting only partial biological overlap.

**Table 1:** Sample characteristics.

	Depressed men			Depressed women		
	No lifetime (hypo)manic episode (N = 209)	Lifetime (hypo)manic episode (N = 56)	p^a^	No lifetime (hypo)manic episode (N = 431)	Lifetime (hypo)manic episode (N = 68)	p^a^
**Sociodemographics**						
Age (years), mean (sd)	43.9 (11.6)	42.7 (11.0)	.49	40.4 (12.4)	38.3 (11.9)	.19
Education (years), mean (sd)	12.0 (3.2)	11.0 (3.0)	.03	12.1 (3.2)	11.4 (3.4)	.10
North-European ancestry, n (%)	203 (97.1)	54 (96.4)	.79	402 (93.3)	63 (92.6)	.85
**Sampling factors**						
Time of awakening, mean (sd)	7:26 (0:59)	7:34 (1:01)	.37	7:25 (1:02)	7:34 (0:58)	.28
Working on day of sampling, n (%)	148 (70.8)	41 (73.2)	.73	274 (63.6)	50 (73.5)	.11
Sampling on weekday, n (%)	198 (94.7)	49 (87.5)	.06	400 (92.8)	66 (97.1)	.19
Sampling in month with more daylight, n (%)	147 (70.3)	40 (71.4)	.87	284 (65.9)	52 (76.5)	.08
**Health factors**						
Smoking status			.61			.11
Never, n (%)	55 (26.3)	12 (21.4)		132 (30.6)	23 (33.8)	
Former, n (%)	68 (32.5)	17 (30.4)		142 (32.9)	14 (20.6)	
Current, n (%)	86 (40.1)	27 (48.2)		157 (36.4)	31 (45.6)	
Alcohol use			.31			.37
No, n (%)	29 (13.9)	12 (21.4)		88 (20.4)	19 (27.9)	
Moderate, n (%)	157 (75.1)	40 (71.4)		290 (67.3)	41 (60.3)	
Heavy, n (%)	23 (11.)	4 (7.1)		53 (12.3)	8 (11.8)	
Physical activity (1000 MET-min/week), mean (sd)	3.6 (3.3)	4.0 (3.5)	.53	3.5 (3.0)	3.6 (3.2)	.77
Sleep (<6 hours)	122 (58.4)	37 (66.1)	.30	239 (55.5)	40 (58.8)	.60
Body Mass Index, mean (sd)	26.05 (4.6)	27.29 (5.1)	.08	25.3 (5.7)	26.1 (5.8)	.27
Cardiovascular disease, n (%)	13 (6.2)	5 (8.9)	.47	19 (4.4)	2 (2.9)	.58
Diabetes, n (%)	11 (5.3)	3 (5.4)	.98	12 (2.8)	1 (1.5)	.53
Number of other chronic diseases, mean (sd)	0.9 (1.0)	1.1 (1.1)	.14	1.0 (1.1)	1.2 (1.2)	.13
Statin use, n (%)	23 (11.)	5 (8.9)	.65	19 (4.4)	2 (2.9)	.58
Anti-inflammatory medication use, n (%)	12 (5.7)	4 (7.1)	.70	22 (5.1)	2 (2.9)	.44
**Cortisol indicators**						
AUCg (nmol/l/h), mean (sd)	19.19 (6.93)	19.27 (6.96)	.96	19.66 (7.43)	19.94 (7.79)	.84
AUCi (nmol/l/h), mean (sd)	1.68 (5.98)	1.22 (8.27)	.73	3.04 (6.15)	1.81 (6.95)	.29
Evening cortisol (nmol/l)^b^, mean (sd)	4.65 (1.79)	4.75 (1.73)	.85	4.93 (1.75)	4.93 (1.10)	.99
Diurnal cortisol slope (decline/h)^b^, mean (sd)	0.73 (0.39)	0.79 (0.50)	.50	0.72 (0.38)	0.72 (0.39)	.92
**Inflammatory markers**						
CRP (mg/l)^b^, mean (sd)	1.11 (5.28)	1.23 (8.57)	.57	1.24 (5.21)	1.15 (5.88)	.79
IL-6 (pg/ml)^b^, mean (sd)	0.75 (4.65)	0.71 (6.41)	.96	0.67 (3.89)	0.78 (3.60)	.34
TNF-α (pg/ml)^b^, mean (sd)	0.68 (4.78)	0.89 (1.56)	.15	0.70 (3.82)	0.88 (3.88)	.16

Abbreviations: sd, standard deviation; AUCg, area under the curve with respect to the ground; AUCi area under the curve with respect to increase; CRP, C-reactive Protein; IL-6, Interleukin-6; TNF-α, Tumor Necrosis Factor Alpha.

^a^ Based on T-tests for continous variables and χ^2^-test for dichotomous and categorical variables.

^b^ To normalize parameters, evening cortisol, CRP, IL-6 and TNF-α were ln-transformed, for interpretation means and standard deviations were back transformed.

**Table 2:** Pearson’s correlations between cortisol indicators and inflammatory markers.

	Cortisol indicators				Inflammatory markers		
*Depressed men*	**AUCg**	**AUCi**	**Even.**	**Diurn.**	**CRP**	**IL-6**	**TNF-α**
**Cortisol indicators**							
AUCg	-						
AUCi	0.40*	-					
Evening cortisol	0.30*	0.11	-				
Diurnal cortisol slope	0.43*	-0.45*	-0.08	-			
**Inflammatory markers**							
CRP	0.02	-0.06	0.04	0.07	-		
IL-6	-0.01	-0.07	0.00	0.06	0.67*	-	
TNF-α	0.00	-0.04	0.17*	0.03	0.31*	0.28*	-
*Depressed women*	**AUCg**	**AUCi**	**Even.**	**Diurn.**	**CRP**	**IL-6**	**TNF-α**
**Cortisol indicators**							
AUCg	-						
AUCi	0.58*	-					
Evening cortisol	0.34*	0.09	-				
Diurnal cortisol slope	0.37*	-0.35*	-0.12*	-			
**Inflammatory markers**							
CRP	-0.07	-0.06	-0.08	-0.05	-		
IL-6	-0.05	-0.03	-0.09	-0.03	0.57*	-	
TNF-α	-0.04	-0.07	-0.01	0.04	0.46*	0.54*	-

Abbreviations: even., evening cortisol; diurn., diurnal cortisol slope; AUCg, area under the curve with respect to the ground; AUCi area under the curve with respect to increase; CRP, C-reactive Protein; IL-6, Interleukin-6; TNF-α, Tumor Necrosis Factor Alpha.

* = p <.05.

Table 3 shows the results of logistic regression analyses separately relating each of the cortisol indicators and inflammatory markers to a lifetime (hypo)manic episode. None of the cortisol indicators and inflammatory markers was significantly associated with a lifetime (hyp)manic episode (i.e. all p≥.15 in depressed men, all p≥.15 in depressed women) and, consequently, analyses on *independent associations* were not further performed.

**Table 3:** Associations of cortisol indicators and inflammatory markers with a lifetime (hypo)manic episode.

	Lifetime (hypo)manic episode Adjusted for basic covariates		
*Depressed men*	**OR**	**95% CI**	**p** ^**a**^
**Cortisol indicators**			
AUCg (nmol/l/h) (N = 168)	1.01	0.95–1.07	.80
AUCi (nmol/l/h) (N = 168)	0.99	0.93–1.06	.86
Evening cortisol (nmol/l) (N = 200)	1.03	0.54–1.97	.93
Diurnal cortisol slope (decline/h) (N = 152)	1.75	0.60–5.11	.31
**Inflammatory markers**			
CRP (mg/l) (N = 266)	1.05	0.87–1.25	.63
IL-6 (pg/ml) (N = 266)	0.99	0.83–1.19	.95
TNF-α (pg/ml) (N = 266)	1.42	0.88–2.27	.15
*Depressed women*	**OR**	**95% CI**	**p** ^**a**^
**Cortisol indicators**			
AUCg (nmol/l/h) (N = 325)	1.00	0.95–1.06	.87
AUCi (nmol/l/h) (N = 325)	0.97	0.91–1.03	.30
Evening cortisol (nmol/l) (N = 381)	0.98	0.56–1.72	.98
Diurnal cortisol slope (decline/h) (N = 306)	0.93	0.32–2.70	.89
**Inflammatory markers**			
CRP (mg/l) (N = 500)	0.96	0.83–1.11	.57
IL-6 (pg/ml) (N = 500)	1.12	0.89–1.40	.33
TNF-α (pg/ml) (N = 500)	1.22	0.93–1.61	.15

Abbreviations: OR, odds ratio; CI, confidence interval, AUCg, area under the curve with respect to the ground; AUCi area under the curve with respect to increase; CRP, C-reactive Protein; IL-6, Interleukin-6; TNF-α, Tumor Necrosis Factor Alpha.

^a^ Based on multivariable logistic regression analyses comparing depressed patients with a lifetime (hypo)manic episode to depressed patients without a lifetime (hypo)manic episode (reference), adjusted for basic covariates (sociodemographics, sampling factors).

To examine *effect modification* between HPA-axis activity and immunological activity in relation to a lifetime (hypo)manic episode, we first tested the interaction terms between all cortisol indicators and inflammatory markers (see Table 4). Diurnal cortisol slope and CRP showed a significant interaction in depressed men (p = .005), whereas significant interactions were found for evening cortisol and IL-6 (p = .049) in depressed women. These significant interactions were further explored by combining information on the level (low versus high) of these specific cortisol indicators and inflammatory markers (see Table 5). Compared to depressed men with low levels of diurnal cortisol slope and CRP, the odds of having a lifetime (hypo)manic episode was significantly increased in men with both high diurnal cortisol slope and CRP levels (OR = 5.23, p = .008, adjusted for basic covariates). After additional adjustment for health factors, these odds increased even more for men with high levels on both diurnal cortisol slope and CRP (OR = 10.99, p = .001). Although the direction and magnitude of the ORs showed some variation in depressed women, no significant differences were found between women with high evening values only (p = .19), high IL-6 levels only (p = .35) and both high evening cortisol and IL-6 levels (p = .50).

**Table 4:** Significance of interactions between cortisol indicators and inflammatory markers in relation to a lifetime (hypo)manic episode^a^.

	Inflammatory markers		
	CRP	IL-6	TNF-α
*Depressed men*	**p**	**p**	**p**
**Cortisol indicators**			
AUCg	.06	.23	.45
AUCi	.17	.50	.77
Evening cortisol	.90	.49	.62
Diurnal cortisol slope	.005	.07	.48
*Depressed women*	**p**	**p**	**p**
**Cortisol indicators**			
AUCg	.28	.40	.60
AUCi	.89	.20	.67
Evening cortisol	.48	.05	.07
Diurnal cortisol slope	.81	.70	.90

Abbreviations: AUCg, area under the curve with respect to the ground; AUCi area under the curve with respect to increase; CRP, C-reactive Protein; IL-6, Interleukin-6; TNF-α, Tumor Necrosis Factor Alpha.

^a^ Based on multivariable logistic regression analyses comparing depressed patients with a lifetime (hypo)manic episode to depressed patients without a lifetime (hypo)manic episode (reference), adjusted for basic covariates (sociodemographics, sampling factors).

**Table 5:** Associations of combined cortisol and inflammatory groups with a lifetime (hypo)manic episode.

	Lifetime (hypo)manic episode Adjusted for basic covariates^a^			Lifetime (hypo)manic episode Additionally adjusted for health factors^b^		
*Depressed men*	**OR**	**95% CI**	**p**	**OR**	**95% CI**	**p**
Slope*CRP						
Low slope / low CRP (N = 63)	*Reference*			*Reference*		
High slope/ low CRP (N = 33)	0.56	0.14–2.31	.42	0.43	0.09–2.07	.29
Low slope / high CRP (N = 33)	0.50	0.12–2.07	.34	0.53	0.10–2.72	.45
High slope/ high CRP (N = 23)	5.23	1.55–17.62	.008	10.99	2.52–47.91	.001
*Depressed women*	**OR**	**95% CI**	**p**	**OR**	**95% CI**	**p**
Evening*IL-6						
Low evening / low IL-6 (N = 145)		*Reference*				
High evening / low IL-6 (N = 95)	1.70	0.76–3.79	.19	-	-	-
Low evening / high IL-6 (N = 63)	1.55	0.62–3.86	.35	-	-	-
High evening / high IL-6 (N = 34)	0.59	0.13–2.76	.50	-	-	-

Abbreviations: OR, odds ratio; CI, confidence interval, AUCg, area under the curve with respect to the ground; AUCi area under the curve with respect to increase; CRP, C-reactive Protein; IL-6, Interleukin-6; TNF-α, Tumor Necrosis Factor Alpha.

^a^ Based on multivariable logistic regression analyses comparing depressed patients with a lifetime (hypo)manic episode to depressed patients without a lifetime (hypo)manic episode (reference), adjusted for basic covariates (sociodemographics, sampling factors).

^b^ Based on multivariable logistic regression analyses comparing depressed patients with a lifetime (hypo)manic episode to depressed patients without a lifetime (hypo)manic episode (reference), additionally adjusted for health factors.

## Discussion

To our knowledge, this was the first study that considered the interrelatedness of the HPA-axis and immune system in a large sample of depressed patients with additional information on the presence of a lifetime (hypo)manic episode. Our study demonstrated that neither cortisol indicators nor inflammatory markers were (independently) associated with a lifetime (hypo)manic episode in the overall analyses in depressed men and women. When exploring possible effect modification, most of the analyses again yielded non-significant results. However, the association between diurnal cortisol slope and a lifetime (hypo)manic episode depended on CRP level, and vice versa, in depressed men. More specifically, depressed men with high levels of both diurnal cortisol slope and CRP had a 5-fold increased odds of having a lifetime (hypo)manic episode compared to men with low levels of both diurnal cortisol slope and CRP. Adjustment for the potentially confounding effects of health factors did even strengthen the association. No significant group differences were found in women.

An important strength of our study was the simultaneous assessment of multiple cortisol indicators and inflammatory markers and our findings, indeed, suggest that combining this information may help to distinguish patients with BD from those with UD. However, some limitations have to be recognized. First of all, we tested 24 interaction terms, and only found 2 significant results and 3 trends. Due to the small sample size of patients with BD, correcting for multiple testing with a standard conservative Bonferroni correction (p <.002) would not have yielded any significant results, whereas a more lenient correction with a false discovery rate lower than 0.2 (p <.008), such as advocated by Benjamini and Hochberg [52], would show that these results remain statistically significant. Although we also found a trend towards significance for the interaction between diurnal cortisol slope and IL-6 (p = .07) as well as AUCg and CRP (p = .06), power is limited. We, therefore, would like to encourage future studies to include larger numbers of patients in order to increase the reliability of findings.

Another important limitation of our study is that the CIDI section on (hypo)manic episodes was only conducted at the follow-up assessments after 2 years (lifetime diagnosis), 4 years (diagnosis since the last interview) or 6 years (diagnosis since the last interview). Information on the lifetime presence of a (hypo)manic episode was, thus, mainly based on retrospective data and may, therefore, be less accurate. Since we combined this information of three follow-up assessments, there is also great variation regarding the time since patients had their last (hypo)manic episode, which could influence our results. We therefore advise other studies to prospectively analyze the onset of (hypo)manic episodes in order to determine whether these biological markers can predict the onset of bipolar disorder. Third, cortisol sampling as well as inflammatory marker determination, took place on a single day, and therefore we could not take into account the day-to-day variability of cortisol secretion [53] or the circadian rhythm of cytokine secretion [54].

Finally, before translating our findings to the clinic, by for example combining assessment of inflammatory markers and cortisol indicators as a diagnostic tool is currently not appropriate. Due to practical limitations as well as the need for further data in this topic, we feel that it is far too early to state this could be the biomarker that can differentiate between unipolar and bipolar depression. However, we do think that our results show that researchers as well as clinicians involved in psychiatric biomarker research should always take into account the fact that these systems influence each.

Our study showed that depressed men with both high diurnal cortisol slope and high CRP levels have an increased odds of having a (hypo)manic episode compared to men with low levels of both measures. This was only evident in men, whereas in women this finding could not be replicated. This could be due to the fact that men seem to respond to stress with greater increase in cortisol compared to women [55]. Since inflammation represents a major stressor of the HPA-axis and vice versa [56], it could be that men are more sensitive to these interacting systems than women. Furthermore, this combination of an overactive HPA-axis and immune system may be characteristic of ‘allostatic overload’, a process in which the adaptive mechanisms of regulatory bodily systems become extreme or inefficient [57]. This allostatic overload is often seen in mood disorders [58] and it has been hypothesized that the level of allostatic load reflects the severity of the disorder. As our findings demonstrated that the HPA-axis and immune system, as represented by the diurnal cortisol slope and CRP, are more severely dysregulated in BD patients than in UD patients, this may imply that BD is more severe than UD. This is in line with previous research showing that BD is associated with higher levels of impairments and disabilities compared to UD [59] and may be indirect support for the hypothesis that progression and worsening of outcomes in BD is caused by a higher allostatic load [60]. One of the key mechanisms that are hypothesized to play an important role in the set point of the allostatic load, is the functioning of the Glucocorticoid Receptor (GR). That is, diminished functioning of the GR is thought to play a role in stress related diseases such as mood disorders [61]. Higher cortisol levels at night with a sharper decline during the day (reflected by a high diurnal slope) as well as a diminished inhibition of inflammatory activation (reflected by the general inflammatory marker CRP) could be consequences. Although our findings seem embedded in previous models, the risk of false positive findings cannot be ruled out, as for example it remains unclear how to interpret the lack of any association with IL-6 and TNF-α.

Another interesting observation was that neither cortisol indicators nor inflammatory markers were (independently) associated with a lifetime (hypo)manic episode in depressed men or women. This confirms findings from Su et al. [62], examining inflammation in bipolar depression, and from Becking et al. [27], using the same NESDA sample to examine inflammation in depressed patients with manic symptoms. Both studies did not find any differences in CRP, IL-6 and TNF-α level between depression subtypes. However, our results may partly contradict the findings of Jabben et al. [26] who showed that high diurnal cortisol slope was associated with bipolar spectrum disorder in the same lNESDA sample. Our findings provide important additional information, as it showed that high diurnal cortisol slope was only associated with a (hypo)manic episode in depressed patients with high CRP levels.

In conclusion, our findings suggest that combining information on the HPA-axis and immune system may help to distinguish depressive episodes in the course of BD versus UD. That is, depressed men with high levels of diurnal cortisol slope as well as CRP had an increased odds of having a lifetime (hypo)manic episode, which may have resulted from a higher allostatic load. If these findings are replicated, this would underline the importance of considering HPA-axis and immunological activity simultaneously and we therefore want to encourage other researchers to include assessments on both systems in their future studies.
